# Chiari-like Malformation and Syringomyelia in Pomeranians: A Longitudinal Study

**DOI:** 10.3390/vetsci12070677

**Published:** 2025-07-18

**Authors:** Mees R. Jansma, Marieke van den Heuvel, Kenny Bossens, Erik Noorman, Michelle Hermans, Paul J. J. Mandigers

**Affiliations:** 1 Department of Clinical Sciences, Faculty of Veterinary Medicine, Utrecht University, 3584 CM Utrecht, The Netherlands; 2 Neurology Service, IVC Evidensia Referral Hospital Arnhem, 6825 MB Arnhem, The Netherlands; 3 Neurology Service, Orion Companion Animal Hospital, 2200 Herentals, Belgium; 4 Dierenkliniek Den Heuvel, 5684 NH Oirschot, The Netherlands

**Keywords:** MRI, disease progression, late onset disease, furosemide

## Abstract

Background: Chiari-like malformation (CM) and syringomyelia (SM) are commonly observed conditions in Pomeranian dogs. Affected dogs may develop clinical signs that significantly impact quality of life. Therefore, it is crucial to select only unaffected dogs for breeding. However, the progression of CM/SM has not been fully elucidated. Dogs that are unaffected or mildly affected may progress to severe SM over time. The primary aim of this study is to investigate the progression of CM/SM through repeated MRI scans. A secondary objective is to evaluate the effect of furosemide treatment on syrinx sizes, given its frequent prescription. Methods: Pomeranians that underwent two CM/SM screenings between 2015 and 2025 were included. CM/SM classifications were assessed, and quantitative syrinx measurements were conducted. Maximum syrinx diameter (MSD) and maximum syrinx-to-spinal cord diameter ratio (MSD/SCD-r) were measured and documented. Dogs were classified based on the progression of SM. Furosemide treatment was documented, and its effect on syrinx size was compared with that in dogs not receiving furosemide. Results: At the time of the second MRI, 39.6% of dogs either developed SM or showed substantial progression, whereas 12.5% demonstrated partial recovery. Of the dogs initially classified as free from SM, 20.7% had developed the condition. A significant increase was observed in both MSD (*p* = 0.0058) and MSD/SCD-r (*p* = 0.0038) between MRI1 and MRI2. Notably, the change in MSD between MRI1 and MRI2 was statistically significantly smaller in dogs treated with furosemide compared to untreated dogs (*p* = 0.030). Conclusions: These findings indicate that syrinx dimensions are dynamic and may fluctuate over time, although a general trend toward progression is observed. Furthermore, furosemide may mitigate the progression of SM.

## Simple Summary

Chiari-like malformation (CM) and syringomyelia (SM) are two common health problems in the Pomeranian dog breed that can affect the dog’s quality of life. To prevent these issues in future generations, it is essential to ensure that only healthy dogs are used for breeding. However, it is not yet clear how these two conditions change over time, which can make breeding selection challenging. Dogs that seem healthy can still develop conditions later in life. This study looked at how CM and SM develop over time using repeated MRI scans. We studied Pomeranians that had two MRI scans between 2015 and 2025. We checked the CM/SM status and measured the size of the fluid-filled cavity (syrinx) in their spinal cord. By the time of the second MRI scan, almost 40% of the dogs had either developed SM or seen their condition worsen. The syrinxes got noticeably larger over time. We concluded that the size of the syrinx can change over time, often getting worse.

## 1. Introduction

Chiari-like malformation (CM) and syringomyelia (SM) are two conditions commonly observed in several small dog breeds [1]. A recent study reported the prevalence of CM and SM in Pomeranians as 54.9% and 23.9%, respectively [2]. CM is characterised by a reduced volume of the caudal cranial fossa and the displacement of the cerebellar vermis into or through the foramen magnum, often accompanied by increased cerebellar volume [3,4,5]. It is considered a skull malformation that results in cerebellar compression and herniation [4]. SM is a condition characterised by the formation of a fluid-filled cavity (syrinx) within the spinal cord. It is thought to result from abnormal cerebrospinal fluid (CSF) flow, which may be caused by one or a combination of factors, including abnormalities such as CM, the obstruction of CSF pathways, pressure changes induced by arterial pulsation, and altered CSF pulse wave propagation within the subarachnoid space [3,4,5,6,7,8]. Brachycephalism has been proposed as a potential underlying cause [9,10,11,12]; however, a recent study demonstrated that SM can also occur in non-brachycephalic dogs [1]. This finding suggests that additional or alternative factors contribute to the development of the condition. In Pomeranians, SM is more frequently observed in dogs with lower body weight [2], but body weight itself is not the determining factor. The smaller the dog breed, the greater the risk of developing CM and/or SM [1]. Additionally, while SM is often observed in conjunction with CM in Cavalier King Charles Spaniels (CKCSs), it can also develop independently of CM [1,2,13]. These findings underscore the complexity and multifactorial nature of SM pathogenesis.

Not all dogs affected by CM and/or SM exhibit clinical signs. When a dog exhibits clinical signs associated with CM, this is referred to as CM-associated pain (CM-P). Signs associated with CM may include vocalisation during rapid postural changes and scratching or rubbing the back of the head, ears or face [3]. In the CKCSs, clinical signs of SM include allodynia, phantom scratching, cervico-torticollis, thoracic limb weakness and spinal weakness [14]. In a recent study investigating the clinical signs of Pomeranians affected by CM and/or SM, the most frequently owner-reported clinical signs (ORCS) included scratching with direct skin contact, rubbing of the head or ears, air licking, spontaneous signs of pain, persistent licking of the front and/or hind paws, and phantom scratching [2]. In the CKCSs, a relationship has been identified between syrinx size (>4 mm) and ORCS [15]. However, in the study by Santifort et al. (2023), which included a cohort of 796 Pomeranians, no statistically significant associations were found between quantitative syrinx measurements and ORCS [2].

Two earlier published studies investigated the effect of age on the presence of SM in the CKCSs. Both identified a significant age-related association, with one study indicating a 45% rise in SM prevalence across various age groups [16,17]. Nevertheless, the progression of SM over time remains incompletely understood. CKCS breeders commonly perform repeated screening to avoid using affected dogs for breeding. A recent study demonstrated that the prevalence of SM can be reduced in the CKCSs when only unaffected dogs are selected as parents [18]. These findings highlight the importance of excluding dogs affected by SM from breeding programs. Determining whether a dog is affected by SM requires screening at an age when results are considered reliable. For this reason, the authors of the CKCS study recommend breeding only with older CKCS dogs to avoid selecting individuals that may develop the condition later in life [18]. Similarly, a recent longitudinal follow-up study in 19 Pomeranians demonstrated that SM can also progress in this breed [19].

The primary aim of this study is to investigate the progression of CM/SM over time using repeated Magnetic Resonance Imaging (MRI). A secondary aim is to assess the effect of furosemide treatment on syrinx sizes. Furosemide is commonly prescribed to reduce CSF production [20,21]; however, to the authors’ knowledge, no standardised studies to date have demonstrated its efficacy in dogs affected by SM.

## 2. Materials and Methods

### 2.1. Inclusion Criteria

All included Pomeranian dogs underwent a tailored MRI to investigate CM/SM between 2015 and 2025 as previously described [2]. Dogs were eligible if they had undergone MRI screening at least twice, with a minimum interval of one year between the first and second scan. Inclusion required that dogs be over 6 months old at the time of the first MRI. Prior to inclusion, owners provided written informed consent. Approval from the Utrecht University animal welfare body was requested but deemed unnecessary, as all dogs were referred for clinical indications.

### 2.2. MRI Studies

MRI studies were performed under general anaesthesia (with individualised anaesthetic protocols) with a high-field MRI scanner at either the IVC Evidensia Referral Hospital Arnhem, The Netherlands (Canon Vantage Elan 1.5T MRI, Otawara-shi, Japan) or the Department of Clinical Sciences, Utrecht University, The Netherlands (Philips Ingenia 1.5T MRI, Eindhoven, The Netherlands), or with a low-field MRI scanner at Dierenkliniek den Heuvel, Best, The Netherlands (Vet-MR Grande, 0.25 Tesla, ESAOTE, Genoa, Italy) or Orion Clinic, Herentals, Belgium (Vet-MR Grande, 0.25 Tesla, ESAOTE, Genoa, Italy).

Whether it was a low-field or high-field MRI scanner, both T1-weighted (T1W) and T2-weighted (T2W) sagittal and transverse images were obtained using the settings described in Supplementary Materials. As differentiation between true syrinx margins and surrounding spinal cord oedema is more challenging on T2W images than on T1W images [22], T1W images were used for measurements when available. The maximum slice thickness was 4 millimetres (mm). To ensure consistency across MRI centres, the scanning protocol (Supplementary Materials) was thoroughly discussed with all participating investigators.

Additionally, owners were asked to return to the same clinic for follow-up examinations to minimise variability related to MRI equipment. For positioning, the dog’s head and neck were extended so that the skull base aligned approximately with the floor of the vertebral canal at C1 and C2, as described previously [2]. MRI examination dates and intervals between scans were recorded. When more than three scans were available, only the first and last scans were evaluated. The first and second MRI are referred to as MRI1 and MRI2, respectively. For both scans, each dog was assessed for CM/SM classification, and quantitative measurements of SM were performed. The presence of CM was determined using sagittal images, while SM was evaluated using both sagittal and transverse images. CM was classified according to the grading system described in previous studies [2,17].

Chiari malformation:

- CM0—No cerebellar herniation or impaction (cerebellar uvula rostral to foramen magnum).
- CM1—Cerebellar impaction (cerebellar uvula on the line of the foramen magnum, no CSF present dorsal to the cervicomedullary junction) and non-rounded shape.
- CM2—Cerebellar herniation (cerebellar uvula caudal to the line of the foramen magnum, no CSF present dorsal to the cervicomedullary junction)
- CM3—Cerebellar herniation resembling CM2, but the posterior part of the cerebellum is herniated and shaped like a tongue.
- CM4—Cerebellar herniation resembling CM2, but the posterior part of the cerebellum is severely herniated and distinctly tongue-shaped.

The occurrence of a pre-syrinx was documented when present. A pre-syrinx was defined as a hyperintense area within the spinal cord on transverse or sagittal T2W images, consistent with what is believed to be interstitial spinal cord oedema [22].

Syrinx localisation and extension relative to the vertebral landmarks were documented using sagittal T1W and T2W images covering at least the region from C1 to T4. These observations were performed by a single observer (PM). Quantitative measurements were conducted by two observers (MJ and PM) when a syrinx was present. The maximum syrinx diameter (MSD) was preferably assessed using T1W images. If T1W images were unavailable for either MRI1 or MRI2, transverse T2W images were used for both time points to ensure consistency. In addition, the spinal cord diameter (SCD) was measured at the level of the MSD to calculate the maximum syrinx diameter-to-spinal cord diameter ratio (MSD/SCD-r). When T2W images were used for MSD measurements due to the absence of T1W images, the MSD/SCD-r was likewise determined using T2W images for both scans. For all quantitative measurements, the arithmetic mean of the two observers’ results was calculated. The presence of imaging artefacts was recorded for all MRI studies. DICOM files were analysed using RadiAnt DICOM Viewer (version 2025.1) and the Horos graphical DICOM software (version 3.3.6) [23,24].

All dogs were SM status classified using the quantitative measurements of both MRI examinations. Dogs who deteriorated over time were categorised as “progressed”, those demonstrating partial improvement were categorised as “partially recovered”, and dogs with no observable change were categorised as “not progressed”. Progression of the disease was determined using one of the following criteria: (a) the MSD increased by more than 0.4 mm between MRI1 and MRI2; (b) MSD/SCD-r increased by more than 0.1; or (c) syrinx localisation and extension, as assessed on sagittal T1W/T2W images, increased by more than two vertebral lengths. Partial recovery was determined using the same criteria but in reverse (MSD decrease of 0.4 mm, MSD/SCD-r decrease of more than 0.1 and/or a decrease in extension by more than two vertebrae). Dogs that did not meet any of these criteria were categorised as “not progressed”.

### 2.3. Clinical Data and ORCS

Signalment and other relevant data were recorded, including age, sex, pedigree, microchip identification number, body weight and country of origin. Case records were reviewed retrospectively. The presence of subsequent ORCS at the time of MRI1 and MRI2 was documented as previously described: phantom scratching, air licking, head shaking, head rubbing, scratching the head, shoulders, or neck with direct skin contact, spontaneous signs of pain, and vocalisation [2,25].

### 2.4. Treatment

If the dog exhibited ORCS in combination with CM and/or SM, owners were advised—following the initial consultation—to administer either amitriptyline (starting dose: 1 mg/kg twice daily (BID)) or gabapentin (starting dose: 10 mg/kg twice daily (TID)) as previously described [25]. The choice of neuropathic pain medication was left to the discretion of the owner, after being informed that both medications were effective but differed in pharmaceutical formulation: amitriptyline as a tablet and gabapentin as a liquid. Furosemide (1 mg/kg BID) was added to the treatment regimen if either (a) a pre-syrinx was identified on sagittal or transverse T2W image, or (b) if the dog had an MSD/SCD-r > 30% at MRI1, unless the owners declined this addition. Amitriptyline and gabapentin do not affect syrinx size, whereas furosemide may reduce CSF production and syrinx progression [20,21]. Particular attention was therefore given to furosemide treatment between MRI1 and MRI2. If treatment information was unavailable for a given dog, that individual was excluded from further analysis evaluating the effect of furosemide on syrinx progression. Dogs with SM at either MRI1 and/or MRI2 were subsequently grouped based on furosemide administration (furosemide treatment vs. no furosemide treatment). Mean MSD, mean MSD/SCD-r, and progression status were compared for both groups.

### 2.5. Exclusion Criteria

Dogs diagnosed with space-occupying lesions in the vertebral canal or skull, as well as MRI studies with artefacts or insufficient image quality that precluded accurate assessment, were excluded.

### 2.6. Data Management and Statistical Analysis

Data were analysed using Microsoft Excel (version 16.99), R (version 4.4.3) and RStudio (version 2025.05.1-513) [26,27,28]. Normal distribution and assumptions of normality were evaluated visually using quantile-quantile plots. Differences in CM classification between MRI1 and MRI2 were tested using the McNemar-Bowker test. Differences in SM classification, presence of a pre-syrinx and the presence of ORCS in dogs that progressed between MRI1 and MRI2 were evaluated using the exact McNemar test. For the study population with ORCS, differences in the presence of ORCS were tested with a regular McNemar test. Changes in MSD and MSD/SCD-r were assessed using the Wilcoxon signed-rank test for all dogs, regardless of treatment, as well as for the subgroup of dogs not treated with furosemide. For dogs with SM at MRI1, a paired *t*-test was used to evaluate differences in MSD and MSD/SCD-r. Confidence intervals (CI) for the proportions of progressed dogs and partially recovered dogs were derived using the binomial distribution. In contrast, CIs for mean MSD and MSD/SCD-r in these groups were derived using the t-distribution. Differences in the proportion of dogs that progressed between treatment groups (furosemide vs. no furosemide) were assessed using Fisher’s exact test. Mean difference in MSD and MSD/SCD-r between treatment groups was compared using a two-sample *t*-test, with equality of variances assessed with an F-test. Differences in age at MRI1 and MRI2, weight at MRI1 and MRI2, and scan intervals between treatment groups were evaluated using two-sample *t*-tests or Welch *t*-tests, depending on whether the variances were equal. Differences in sex between groups were assessed using Fisher’s exact test. A *p*-value < 0.05 was considered statistically significant.

## 3. Results

### 3.1. Study Population

A total of 49 dogs were included in the study. Three dogs were scanned on three occasions. One dog was excluded from analysis after a brainstem tumor was identified on MRI2. Of the 48 remaining dogs, 46 were Pomeranians, and two were Pomchí’s (cross Pomeranian-Chihuahua) but exhibited all the phenotypic characteristics of a Pomeranian. An equal sex distribution was observed in the study population, comprising 24 males (50%) and 24 females (50%). The mean ± standard deviation (SD) age of the dogs at MRI1 was 2.1 years ± 1.1 (median: 2.0; range: 0.6–5.3), and at MRI2 was 4.7 years ± 1.4 (median: 4.9; range: 2.4–8.4). The mean ± SD scan interval was 2.6 years ± 1.0 (median: 2.4; range: 1.0–5.8), resulting in the majority of dogs being older than 3 years at MRI2. Body weight data were available for 29 dogs at MRI1 and 38 dogs at MRI2. The mean body weight ± SD at MRI1 was 3.11 kg ± 0.86 (median: 3.10; range: 1.90–5.70) and at MRI2 was 3.59 kg ± 0.93 (median: 3.50; range: 2.10–6.80).

Thirty-three dogs had a pedigree, while the remaining 15 dogs were either not registered at any national kennel club or had no documentation of their pedigree. Thirteen of these 15 dogs descended from pedigree Pomeranians, while two were crossbreeds with a Chihuahua. These two dogs were, based on their appearance, Pomeranians and therefore included. All dogs originated from one of the following countries: Russia (20 dogs, 42%), Netherlands (18, 38%), Belarus (1 dog, 2%), Belgium (1 dog, 2%), Bulgaria (1 dog, 2%), France (1 dog, 2%), Italy (1 dog, 2%) and Kyrgyzstan (1 dog, 2%). The country of origin was unknown for 4/48 dogs (8%).

### 3.2. MRI Studies

CM/SM classifications for both MRI1 and MRI2 are shown in Table 1 and Table 2. A total of 15 dogs showed a change in CM grade between scans, with a decrease observed in four dogs and an increase in eleven dogs (*p* = 0.0396). Six dogs (20.7% without SM at MRI1) developed SM between MRI1 and MRI2, with no dogs exhibiting a decrease in SM grading (*p* = 0.03125). In total 6/48 dogs had a pre-syrinx at MRI1, and 7/48 dogs had a pre-syrinx at MRI2, with no statistically significant difference between time points (*p* = 1.00).

**Figure 1:**
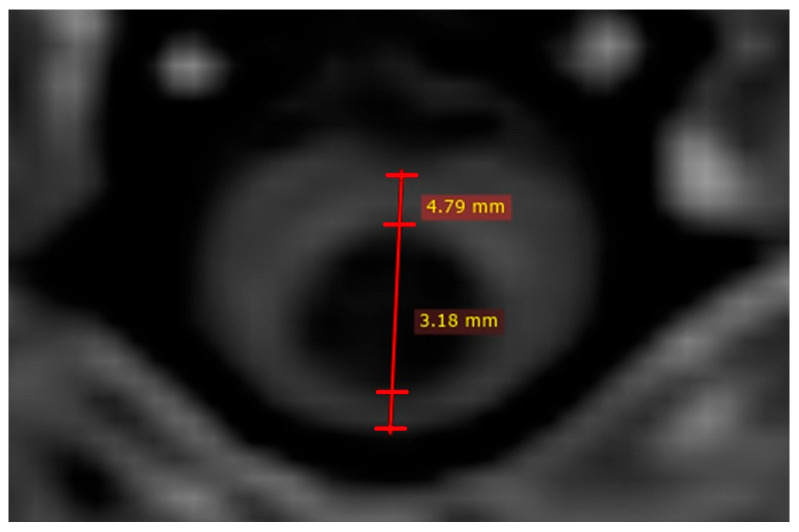
Quantitative syrinx measurements on T1W image. Two measurements are performed; one to assess syrinx size (in this case 3.18 mm) and other to assess spinal cord diameter (in this case 4.79 mm).

Quantitative measurements were performed for 48 dogs at MRI1 and MRI2. Figure 1 provides an example of the measurement technique. T1W images were used to assess the MSD and MSD/SCD-r in 45/48 dogs, while T2W images were used in the remaining 3/48 dogs. The mean MSD ± SD at MRI1 was 0.99 mm ± 1.52 (median: 0; range: 0.00–5.06) and at MRI2 was 1.35 mm ± 1.57 (median: 0.90; range: 0.00–5.53). The mean difference between the MSD ± SD at MRI1 and MRI2 for the overall population (regardless of treatment) was 0.36 mm ± 0.83 (median: 0.00; range: −1.11–2.40), which was statistically significant (*p* = 0.0058; CI = 0.180–1.140) (Table 3). The mean MSD/SCD-r ± SD for the overall population (regardless of treatment) was 16.34% ± 23.17% at MRI1 (median: 0%; range: 0–70.01%) and 22.70% ± 24.70% at MRI2 (median: 18.46%; range: 0–74.99%). The mean ± SD within-individual difference in MSD/SCD-r between MRI1 and MRI2 was 6.36% ± 13.67% (median: 0%; range: −15.35–45.05%), which was statistically significant (*p* = 0.0038; CI= 4.76–19.49%) (Table 3). When considering only the 33 dogs that did not receive furosemide treatment, a similarly significant difference in both MSD and MSD/SCD-r was observed (Table 4). Seven dogs had SM at MRI1 and did not receive furosemide treatment. In this subgroup, neither the mean MSD (*p* = 0.083; CI: −0.12–1.48) nor the mean MSD/SCD-r (*p* = 0.270; CI: −7.14–21.24%) differed significantly between MRI1 and MRI2. However, they did show a non-significant trend towards an increase in syrinx size.

A total of 19/48 dogs were classified as “progressed” (39.58%, CI: 25.77–54.73%). Of these, 17 were categorised based on an increase in syrinx size of at least 0.4 mm, and two dogs exhibited an increase in syrinx extension of at least two vertebral lengths. For the dogs that progressed, the mean ± SD difference in mean MSD and MSD/SCD-r between MRI1 and MRI2 was 1.13 mm ± 0.78 (median: 0.84; range: −0.205–2.40; CI: 0.75–1.50) and 18.03% ± 14.72% (median: 14.53%; range: −8.67–45.05%; CI: 10.94–25.12%), respectively.

A total of 6/48 dogs were classified as “partially recovered” (12.50%; CI: 4.73–25.25%). Five were labelled as such due to a decrease in syrinx size of at least 0.4 mm, and one dog demonstrated a decrease in syrinx extension of at least two vertebrae. For the dogs that partially recovered, the mean ± SD difference in MSD and MSD/SCD-r between MRI1 and MRI2 was −0.72 mm ± 0.28 (median: −0.77; range: −1.11 to −0.33; CI: −1.02 to −0.42) and −6.20% ± 9.17% (median: −10.51%; range: −15.35% to 8.54%; CI: −15.82–3.43%), respectively.

### 3.3. Clinical Data and ORCS

Data regarding ORCS were collected at MRI1 and/or MRI2. Three dogs were excluded from the ORCS analysis due to comorbidities (otitis externa and otitis media) at MRI2. ORCS data was available for 47/48 dogs at MRI1 and 35/48 dogs at MRI2. At the time of MRI1 28/47 dogs (59.57%) exhibited ORCS. Between MRI1 and MRI2, eight dogs developed ORCS, while 5 dogs who initially showed ORCS at MRI1 no longer exhibited them at MRI2. These changes in ORCS were not statistically significant (*p* = 0.405). Among the 19 dogs classified as “progressed”, ORCS were available for 13/19 individuals. Of these, 5/19 dogs developed ORCS between MRI1 and MRI2, while 2/19 dogs no longer exhibited ORCS at MRI2 despite having ORCS at MRI1. These changes in ORCS were not statistically significant (*p* = 0.45).

### 3.4. Furosemide Treatment

Treatment records were available for 45/48 dogs. At the time of MRI1, each dog received a customised medication plan based on its clinical presentation and consultation with the owner. By the time of MRI2, treatment regimens varied across the population, with some dogs receiving a variety of medications that were not initially prescribed. The different administered medications are provided in Table 5. 

**Figure 2:**
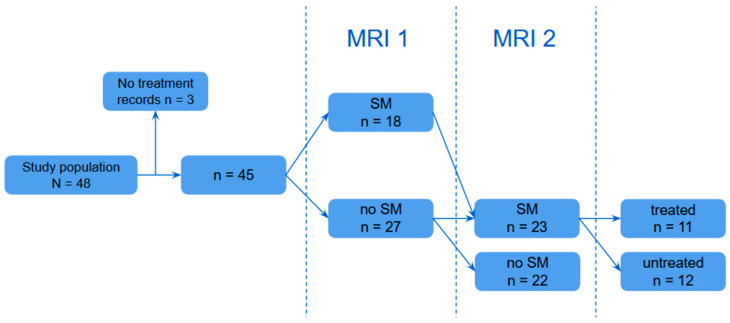
Visual representation of group allocation based on furosemide treatment.

Eleven dogs with SM received furosemide treatment between MRI1 and MRI2, with dosages ranging from 1.04–2.86 mg/kg. In one case, the dosage was increased from 0.6 mg/kg to 1.21 mg/kg between the scans. Dogs classified with SM at either MRI1 and/or MRI2 were subsequently grouped according to furosemide treatment (treated vs. untreated) (Figure 2). The proportion of dogs classified as “progressed” and the changes in MSD and MSD/SCD-r between MRI1 and MRI2 were then compared between these groups. No significant differences were identified between the groups in terms of age, scan interval, weight or sex at either MRI time point. Table 6 summarises the proportion of progressed dogs and mean differences in MSD and MSD/SCD-r between MRI1 and MRI2 for treated versus untreated dogs. The change in MSD between MRI1 and MRI2 was significantly smaller in dogs treated with furosemide compared to those not treated (*p* = 0.030; CI: 0.10–1.74). However, the changes in MSD/SCD-r (*p* = 0.159; CI: −4.30–24.63%) and the proportion of progressed dogs (*p* = 0.069; CI OR: 0.00–1.43) did not differ significantly between the groups. Of the six dogs classified as “partially recovered”, four had received furosemide treatment, one had not, and one lacked treatment records.

## 4. Discussion

This longitudinal study shows a statistically significant difference in both CM and SM classifications between MRI1 and MRI2, with a minimum interval of at least one year. These findings contrast with those reported by Santifort et al. (2024), who observed no significant changes in CM or SM classification over time. It is possible that the absence of significant findings in that study reflected a type II error, as the sample size was smaller (n = 19) [19]. In contrast, Santifort et al. (2023) observed that dogs with CM and/or SM were significantly older than those without, and that dogs over 1.5 years of age were 1.5 times more likely to have CM and 3.2 times more likely to have SM compared to younger dogs [2]. These results align with the findings of the present study. The prevalence of SM is difficult to determine due to its late onset and often not randomly chosen study populations. Previous studies have reported a prevalence of 23.9% in Pomeranians [2], between 27–46% in the well-studied CKCSs and 37.5% in the Griffon Bruxellois [13,18]. It is also considered common in several other toy breeds, including the Chihuahua and Affenpinscher [9].

Several factors may contribute to the observed difference in CM grading between MR1 and MRI2. One possible explanation is variation in head and neck positioning. Flexion of the neck has been reported to increase cerebellar herniation [29]. Thus, differences in positioning between MRI1 and MRI2 could have influenced CM classification grade. Another potential factor is the cardiac cycle, which in humans is known to cause minor cerebellar movements (<1 mm) between systole and asystole [30]. Whether such micro-movements contributed to the variation observed in this study remains unclear. A third consideration relates to the relatively mild manifestation of CM in Pomeranians. The majority of Pomeranians are classified as CM0 or CM1 [2], whereas the majority of CKCSs are classified as CM2 or CM3 [17,18]. Although the CM classification has been well described, it lacks the precision of quantitative metrics such as syrinx measurements. Consequently, subtle cases may be more challenging to classify consistently, especially in the presence of minor differences in positioning between scans. Moreover, previous studies have yielded inconsistent findings regarding changes in CM classification within the CKCSs. Driver et al. (2012) reported a significant increase in cerebellar herniation affecting 73% of CKCS dogs [31], while Wijnrocx et al. (2017) found no significant differences in CM classification between age groups [17]. It remains uncertain whether the significant change in CM classification observed in the present study reflects genuine pathological progression or is primarily attributable to positioning, cardiac cycle or lack of quantitative metrics.

Parker et al. (2011) and Wijnrocx et al. (2017) showed a significant increase in SM prevalence among older CKCSs, with Parker et al. (2011) observing a 1.3-fold increase in odds ratio of SM per additional year of age in asymptomatic dogs [16,17]. Similarly, longitudinal studies by Driver et al. (2012) and Cerda-Gonzalez et al. (2016) demonstrated the onset of SM in multiple CKCS dogs between MRI1 and MRI2 [31,32]. The findings of the present study support these observations by showing that SM can also develop at later ages in Pomeranian dogs. This has important clinical implications, particularly for breeding practices. Breeders often prefer to mate dogs at a young age to reduce the risk of dystocia. However, this approach may inadvertently lead to the selection of individuals who develop SM later in life. The current findings highlight this risk and support the importance of age-appropriate screening in breeding decisions.

This study also identified an increase in both absolute and relative quantitative syrinx measurements between MRI1 and MRI2. Overall, 39.58% of the study population were classified as “progressed”, while 12.5% showed partial recovery. These findings are consistent with the longitudinal study of Santifort et al. (2024) in Pomeranians, which also reported increases in quantitative syrinx measurements over time [19]. Similar trends have been observed in CKCSs: Cerda-Gonzalez et al. (2016) reported this, while Driver et al. (2012) noted a significant increase in SM lesion size [31,32], and Wijnrocx et al. (2017) demonstrated a rise in SM grading in older dogs [17]. However, disease progression is not uniform across individuals. In the present study, 47.92% of dogs did not develop SM during the study period. Moreover, dogs that were diagnosed with SM at MRI1 who did not receive furosemide treatment demonstrated a non-significant trend toward increased syrinx size. Further research is necessary to assess the progression of SM and syrinx size following initial diagnosis.

Furosemide has been proposed as a treatment option for SM, based on its ability to lower CSF production and decrease intracranial pressure [3,20,21,33]. Other medications given during the study period included amitriptyline and gabapentin, which do not affect syrinx size. In the present study, the change in mean MSD differed significantly between dogs with SM that were treated with furosemide and those that were not. Although other outcome measures did not reach statistical significance, they showed potentially meaningful trends. Dogs treated with furosemide exhibited smaller increases in absolute and relative syrinx size, and a lower proportion of dogs were classified “progressed”. Additionally, the majority of dogs that were classified “partially recovered” had received treatment with furosemide. However, due to the small sample size of this subgroup, statistical analysis was not feasible. Evidence supporting the effectiveness of furosemide in the treatment of SM is scarce. A case series in CKCS dogs reported that furosemide neither prevented further syrinx expansion or reduction in syrinx size but acknowledged that its potential role in delaying disease progression could not be excluded [34]. The present study observed syrinx size reduction in both groups. Nonetheless, the significant difference in MSD and the non-significant trends observed in the other parameters suggest that furosemide may contribute to delaying syrinx expansion.

Several limitations must be considered when interpreting the results of this study. Ideally, dogs would be monitored without receiving treatment during the study period; however, withholding treatment was not ethically justifiable for dogs presenting with severe clinical signs. In addition, increased intervals between MRI1 and MRI2 may yield more pronounced results. Although both owners and investigators aimed for a follow-up period of no more than three years, the naturalistic multi-centred study design resulted in variability in scan intervals, age at imaging, and disease stage at evaluation. In three dogs, only T2W transverse images of the spinal cord were obtained. These three dogs were included because a previous study demonstrated a strong correlation between T1W- and T2W-based measurements [2]. Furthermore, treatment allocation could not be randomised. As a result, dogs were evaluated at different stages, which may have influenced the progression of quantitative measurements. The method of inclusion could have caused a selection bias, as dogs that deteriorated clinically may have been more likely to undergo a second MRI. Conversely, survivor bias may also be of concern, as only dogs that were not euthanised as a result of CM/SM progression before the second MRI were included. As earlier explained, reducing variance in positioning proved to be challenging. To address this, the scanning protocol was thoroughly discussed with all participating institutions, and owners were requested to return to the same clinic for follow-up imaging to reduce inter-centre variability. Nevertheless, it cannot be excluded that these small-sized dogs were positioned differently between the two MRIs. A final limitation of this study is that the MRI protocol used does not include post-contrast images, which are useful for investigating, for example, a case with meningoencephalomyelitis. However, it is very unlikely that we accidentally included dogs with such a disease, as none of the medications used were suitable to treat a meningoencephalomyelitis. Moreover, it is unlikely that a dog with such a condition would go unnoticed, considering the follow-up period was very long.

## 5. Conclusions

The present study demonstrates a significant increase in SM classifications over time. Notably, 20.7% of dogs initially without SM developed the condition by the time of MRI2. In total, 39.58% of dogs either developed SM or progressed substantially, while 12.5% of dogs showed partial recovery. Both absolute and relative quantitative syrinx measurements increased significantly between MRI1 and MRI2. In dogs that presented with SM at MRI1, a statistically non-significant trend was observed regarding an increase in syrinx size. These findings suggest that syrinx sizes are dynamic and can fluctuate over time, though they generally show a progressive trend. Finally, the findings suggest that furosemide may help slow the progression of SM. However, further research is needed to prove its effectiveness.

## Figures and Tables

**Table 1:** CM classifications at MRI1 and MRI2, including the *p*-value * for the dogs with different CM classifications at MRI1 and MRI2.

Classification	CM0 MRI2	CM1 MRI2	CM2 MRI2	*p*-Value
**CM0 MRI1**	6	10	1	
**CM1 MRI1**	2	25	0	
**CM2 MRI1**	0	2	2	
***p*** **-value**				0.04 *

* No dogs were classified as CM3 and CM4. Therefore, these scores were not included in the table.

**Table 2:** SM classification at MRI1 and MRI2, including the *p*-value * for the dogs with different SM classification at MRI1 and MRI2.

Classification	No SM MRI2	SM MRI2	*p*-Value
**No SM MRI1**	23	6	
**SM MRI1**	0	19	
***p*** **-value**			0.03 *

**Table 3:** Changes in mean MSD and MSD/SCD-r between MRI1 and MRI2 for all 48 dogs, regardless of whether they received furosemide. Asterisk (*) indicates statistical significance.

Parameter	MRI1	MRI2	*p*-Values
**MSD**	0.99 mm ± 1.52	1.35 mm ± 1.57	0.0058 *
**MSD/SCD-r**	16.34% ± 23.17%	22.70% ± 24.70%	0.0038 *

**Table 4:** Changes in mean MSD and MSD/SCD-r between MRI1 and MRI2 for 33 dogs that did **not** receive furosemide treatment. Asterisk (*) indicates statistical significance.

Parameter	MRI1	MRI2	*p*-Values
**MSD**	0.42 mm ± 0.96	0.82 mm ± 1.28	0.0047 *
**MSD/SCD-r**	7.95% ± 17.57%	14.02% ± 21.06%	0.0150 *

**Table 5:** Number of dogs receiving different medical treatment between MRI1 and MRI2.

Type of Medication	Number of Dogs
Amitriptyline	12
Bedinvetmab	1
Buprenorphine	1
Cannabinoid oil	6
Furosemide	12
Gabapentin	3
NSAID	4
Oclacitinib	2
Pregabalin	2
Tramadol	2

**Table 6:** Mean differences in MSD ± SD and MSD/SCD-r, and proportion of progressed dogs between MRI1 and MRI2 for dogs suffering from SM treated with or without furosemide. An asterisk (*) indicates statistical significance.

Treatment Groups	MSD	MSD/SCD-r	Proportion Progressed
Treated (n = 11)	0.185 mm ± 0.959 (range: −1.11–2.29)	6.54% ± 15.29% (range: −10.91–45.05%)	54.55% (CI: 23.38–83.25%)
Untreated (n = 12)	1.104 mm ± 0.934 (range: −0.78–2.40)	16.70% ± 17.83% (range: −15.35–39.78%)	91.67% (CI: 62.52–99.79%)
***p*** **-value and CI**	0.030 * (CI: 0.10–1.74)	0.159 (CI: −4.30–24.63%)	0.069 (CI OR: 0.00–1.43)

## Data Availability

The raw data supporting the conclusions of this article will be made available by the authors, if requested.

## Supplementary Materials

The following supporting information can be downloaded at: https://www.mdpi.com/article/10.3390/vetsci12070677/s1.

## Abbreviations

The following abbreviations are used in this manuscript:

BID	Twice daily
CI	Confidence interval
CKCS	Cavalier King Charles Spaniel
CM	Chiari-like malformation
CM-P	CM-associated pain
CSF	Cerebrospinal fluid
mm	Millimetres
MRI	Magnetic Resonance Imaging
MSD	Maximum syrinx diameter
MSD/SCD-r	Maximum syrinx diameter/spinal cord diameter ratio
ORCS	Owner reported clinical signs
SCD	Spinal cord diameter
SD	Standard deviation
SM	Syringomyelia
T1W	T1-weighted image
T2W	T2-weighted image
TID	Thrice daily
