# Supplementary material for: Chiari-like Malformation and Syringomyelia in Pomeranians: A Longitudinal Study
Source: Vet Sci. 2025 Jul 18;12(7):677. doi: 10.3390/vetsci12070677 (PMC12299236; doi:10.3390/vetsci12070677)
Supplement: Supplementary file 1 [file vetsci-12-00677-s001.zip › vetsci-3752039-supplementary.pdf]

veterinary sciences

Supplementary Materials

The following sequences were minimally acquired to ensure diagnostic-quality MRI for the assessment of Chiari-like malformation (CM) and syringomyelia (SM) in this study:

- Sagittal T1-weighted (T1W) and T2-weighted (T2W) images must extend from the interthalamic adhesion to at least T4/T5 intervertebral disc space, with a maximum slice thickness of 4mm. The head and neck should be aligned in the sagittal plane. If complete alignment was not feasible, separate sagittal images of the brain and cervical spinal cord must be acquired to ensure midline sagittal representation of each structure. Images were considered non-diagnostic if the central nervous system (CNS), from the cisterna magna to the T4/T5 intervertebral disc space, was not visible in a single continuous sagittal image. An exception applies in case of scoliosis, where dorsal plane images of the affected region must also be provided. Scoliosis associated with SM typically co-occurs with a marked syrinx. In the absence of syringomyelia, the presence of scoliosis may be questioned and the images deemed insufficient.
- Transverse T1W or T2W images should be obtained perpendicular to the spinal cord at the level of the maximum syrinx width, if syringomyelia (SM) is present. Alternatively, in the absence of a syrinx, a transverse image block should be centered at the C3 vertebra, extending from at least the midpoint of the vertebral body of C2 to the midpoint of the vertebral body of C4. The maximum slice thickness was 4 mm.
- Positioning requirements: The head and neck should be positioned in extension such that the skull base is approximately aligned with the floor of the vertebral canal through C1 and C2.

 https://doi.org/10.3390/vetsci12070677
